# Sex Differences in Characteristics and Outcomes among Low-Risk Non-ST-Elevation Acute Coronary Syndrome Patients during Long Term Follow-Up

**DOI:** 10.3390/jcm10132802

**Published:** 2021-06-25

**Authors:** Ivica Kristić, Andrija Matetic, Nikola Crnčević, Frane Runjić, Ozren Polašek, Mislav Vrsalovic

**Affiliations:** 1 Department of Cardiology, University Hospital of Split, 21000 Split, Croatia; kristicivica@gmail.com (I.K.); andrija.matetic@gmail.com (A.M.); ncrncev1@gmail.com (N.C.); frane.runjic@gmail.com (F.R.); 2 Department of Public Health, University of Split School of Medicine, 21000 Split, Croatia; opolasek@gmail.com; 3 Department of Cardiology, Sestre Milosrdnice University Hospital Center, Vinogradska cesta 29, 10000 Zagreb, Croatia; 4 Department of Internal Medicine, University of Zagreb School of Medicine, Šalata 3b, 10000 Zagreb, Croatia

**Keywords:** sex, non-ST-elevation acute coronary syndrome, long-term follow-up

## Abstract

Previous heterogenous studies show conflicting data about sex-based outcomes of non-ST-elevation acute coronary syndrome (NSTE-ACS) patients. This study evaluated 300 NSTE-ACS patients undergoing a coronary angiography between September 2012 and May 2015 that were managed with all-treatment strategies. The sample was stratified by sex and analyzed for the baseline characteristics and outcomes. The main outcome included major adverse cardiovascular and cerebrovascular events (MACCE), which were a composite of cardiac death, nonfatal myocardial infarction, ischemic stroke or urgent coronary revascularization. The female patients were older (median of 69.0 vs. 63.0 years, *p* = 0.008) and had lower values of BMI (median of 26.3 vs. 28.2 kg/m^2^, *p* < 0.001) and eGFR (76.44 ± 22.43 vs. 94.04 ± 27.91 mL/min, *p* < 0.001). There was no significant difference in the treatment strategies, angiographic characteristics and discharge therapy between the groups (*p* > 0.05). The female patients had significantly higher unadjusted rates of ischemic stroke (4.2% vs. 0.5%, *p* = 0.023), cardiac mortality (11.3%, vs. 3.9%, *p* = 0.022) and MACCE (33.8%, vs. 19.5%, *p* = 0.014); female sex was a significant predictor of MACCE in the univariate analysis (HR 1.86, 95%CI 1.12–3.09, *p* = 0.014); and the cumulative incidence of MACCE was higher in female patients (*p* = 0.014). After the adjustment, the predictive effect of female sex became non-significant (HR 1.60, 95%CI 0.94–2.73, *p* = 0.083), while there was no difference in the cumulative incidence of MACCE among the propensity score matched cohort (*p* = 0.177). Female NSTE-ACS patients have worse long-term outcomes compared to their male counterparts. However, the differences disappear after adjustment and propensity score matching. Continuing efforts and health measures are required to alleviate any sex-based differences in the NSTE-ACS population.

## 1. Introduction

Non-ST-elevation acute coronary syndrome (NSTE-ACS) is one of the leading causes of cardiovascular morbidity and mortality [1,2]. As such, it still represents a global health burden with substantial health consequences [2,3]. However, disparities in the management and outcomes of this heterogenous patient group still exist irrespective of the established measures and efforts by international societies [1].

Initial studies reported sex-related inequalities, with female NSTE-ACS patients having worse management and outcomes [4,5,6,7]. Female and male patients have different anatomical and physiological characteristics, including neurohormonal status, body fat composition, vascular anatomy and psychological factors [8]. Furthermore, female patients often have an atypical presentation and lower troponin levels, leading to underdiagnosis, longer emergency department stays and a lower number of admissions to coronary care units [7,9,10,11]. A poor medical sensibility and a higher prevalence of non-atherosclerotic conditions (Takotsubo syndrome, spontaneous coronary artery dissection (SCAD), etc.) could also affect the care of female patients [9,10]. Additional major drivers of undesirable events in the female population are baseline differences, including older age and higher comorbidity burden. Finally, the underuse of invasive management and guideline-directed medication therapy have been largely reported in female patients [10,12].

Therefore, emerging multiple adjusted analyses report against a sex-related disparity among the NSTE-ACS population. Yet, the heterogeneity of studies, population diversity and somewhat conflicting data prevent us from drawing contemporary conclusions. Fluctuation in the follow-up duration, underutilization of coronary angiography and different management strategies are some of the rationales for additional studies. In addition, the lack of systematic risk stratification impairs the comparison of different studies [13]. This has been emphasized in the latest NSTE-ACS guidelines to support further research, diminish any sex-based differences and provide adequate healthcare measures [1].

Therefore, this study enrolled NSTE-ACS patients who completely underwent invasive coronary angiography and were managed with all-treatment strategies (percutaneous coronary intervention (PCI), coronary artery bypass grafting (CABG) or conservative management). The aim of this study was to investigate sex-based differences in the characteristics and long-term outcomes of NSTE-ACS patients, while controlling for any potential confounding effects. Additionally, the aim of the study was to determine the prognostic strength of the GRACE risk score in different sex categories.

## 2. Materials and Methods

### 2.1. Ethical and Institutional Considerations

This study was conducted in accordance with the ethical standards and amendments of the Declaration of Helsinki. All subjects were informed about the goal and course of this study. Written informed consent for coronary angiography and informed consent for the use of relevant medical data has been obtained from all participants. The Ethical Committee of the University Hospital of Split, Croatia (No. 2181-147-01/06) has approved the study protocol.

### 2.2. Subjects and Study Design

This single center observational prospective study enrolled a total of 300 eligible NSTE-ACS patients who underwent a coronary angiography at the University Hospital of Split between September 2012 and May 2015. Patients with active malignant disease and a history of CABG were not considered eligible for the study. A total of 24 patients were lost in follow-up due to their refusal of a further follow-up or loss of adequate communication (Supplementary Figure S1). All patients were followed up through strictly scheduled clinical visits or telephone interviews firstly 3 months after the index event and thereafter at a 12-month interval, with a final contact in May 2017. The diagnosis of NSTE-ACS was established according to the relevant international guidelines [1,14].

### 2.3. Clinical Assessment

Study data were collected using the patient medical records, including electrocardiograms, laboratory reports, procedural data, and angiographic results during index hospitalization. Anthropometric data were collected according to the standard methods. 

### 2.4. Treatment Strategies

All patients underwent an invasive coronary angiography, followed by the appropriate treatment, including PCI, CABG or conservative management. Treatment strategy was determined by at least 2 interventional cardiologists on a case-to-case basis with respect to the patient’s preferences. Patients with multivessel coronary artery disease or technically challenging lesions were presented to the Heart Team involving cardiac surgeons and interventional cardiologists. The radial approach for an invasive coronary angiography was used in most cases. Irrespective of the treatment strategy, all patients received pharmacological therapy with 12 months of dual antiplatelet therapy, according to the relevant guidelines.

### 2.5. Aims and Outcomes

The main aim of the study was to evaluate the differences between sex categories in baseline characteristics and study outcomes. An additional aim was to determine the predictive strength of the GRACE score in each sex category. Primary outcome included major adverse cardiovascular and cerebrovascular events (MACCE), which were a composite of cardiac mortality, nonfatal acute myocardial infarction (AMI), ischemic stroke or urgent coronary revascularization. Secondary outcomes included cardiac mortality, nonfatal AMI, ischemic stroke and urgent coronary revascularization. Each outcome was carefully evaluated by a team of experienced cardiology specialists (I.K., F.R. and M.V.) before the inclusion. Cardiac mortality encompassed each death without sufficient evidence of another non-cardiac cause of death. Nonfatal AMI was defined as a recurrent AMI with or without ST-elevation, according to the relevant guidelines. Unstable angina was also only included among nonfatal AMI events if there was an angiographic confirmation of an unstable lesion and a subsequent revascularization. Urgent coronary revascularization was defined as an urgent intervention, percutaneous or surgical, due to highly symptomatic stable angina.

### 2.6. Statistical Analysis

Statistical analysis was conducted according to standard statistical methods. The normality of data distribution was assessed using the Kolmogorov–Smirnov test. Continuous data were presented as mean ± standard deviation (SD) or as median (interquartile range, IQR). Student’s *t*-test or Mann–Whitney U test were used for continuous data analysis according to parametric or non-parametric distribution, respectively. Categorical variables were expressed as numbers and percentages and analyzed using the chi-squared test. The cumulative incidence of MACCE was estimated using the Kaplan–Meier approach, and the significance was assessed using the Mantel–Cox log-rank test. Cox logistic regression analysis was performed to determine the predictors of MACCE in the multivariate model. The multivariable model included variables that were significantly different between the groups or were previously shown to potentially influence the outcomes (sex, age, BMI, LVEF, eGFR). The results of the risk analyses are provided as hazard ratios (HRs) and 95% confidence intervals (95% CI) that corresponds to a 1-unit increase/decrease in each score on a continuous scale. The accuracy of the GRACE score MACCE prediction was determined for each sex category using the receiver operating characteristic (ROC) and area under the curve (AUC). Finally, a propensity score matching analysis was used to account for the confounding variables and control the selection bias. It has been conducted using a SPSS plug-in for R with a nearest-neighbor matching algorithm and a 1:1 ratio, while a caliper of 0.01 has been set to improve the matching criteria. A two-sided *p*-value of <0.05 was considered significant. Statistical data analysis was carried out using Statistical Package for the Social Sciences (SPSS) software (IBM Corp, NY, USA; version 20).

## 3. Results

A total of 300 patients were initially enrolled in the study protocol, while 276 completed the follow-up, including 71 female and 205 male patients. The female patients were older (median of 69.0 vs. 63.0 years, *p* = 0.008) and had lower values of BMI (median of 26.3 vs. 28.2 kg/m^2^, *p* < 0.001). When looking at the laboratory parameters, the female patients had lower values of Hgb (132.13 ± 13.39 vs. 144.39 ± 14.86 g/L, *p* < 0.001) and eGFR (76.44 ± 22.43 vs. 94.04 ± 27.91 mL/min, *p* < 0.001). Importantly, there was no difference in the follow-up duration between the female and male patients (median of 30.1 vs. 33.1 months, *p* = 0.083, respectively). The other baseline characteristics are presented in Table 1.

There was no significant difference in the treatment strategies, angiographic characteristics and discharge therapy between the sex groups (Table 2).

**Figure 1:**
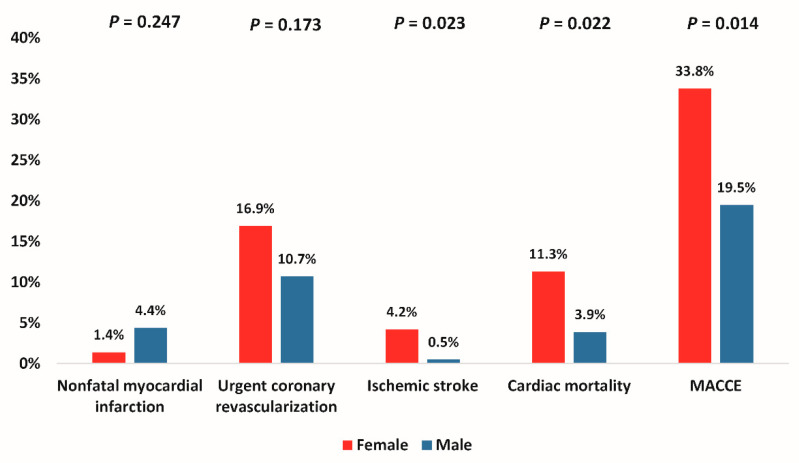
Adverse events during the study period. Legend: MACCE, major adverse cardiovascular and cerebrovascular events (composite of cardiac mortality, ischemic stroke, urgent coronary revascularization and nonfatal myocardial infarction).

The female patients had significantly higher unadjusted rates of ischemic stroke (4.2%, *n* = 3 vs. 0.5%, *n* = 1, *p* = 0.023) and cardiac mortality (11.3%, *n* = 8 vs. 3.9%, *n* = 8, *p* = 0.022) during the follow up, while there was no difference in the rates of nonfatal myocardial infarction and urgent coronary revascularization. Importantly, MACCE occurred significantly more often in the female group (33.8%, *n* = 24 vs. 19.5%, *n* = 40, *p* = 0.014) (Figure 1).

**Figure 2:**
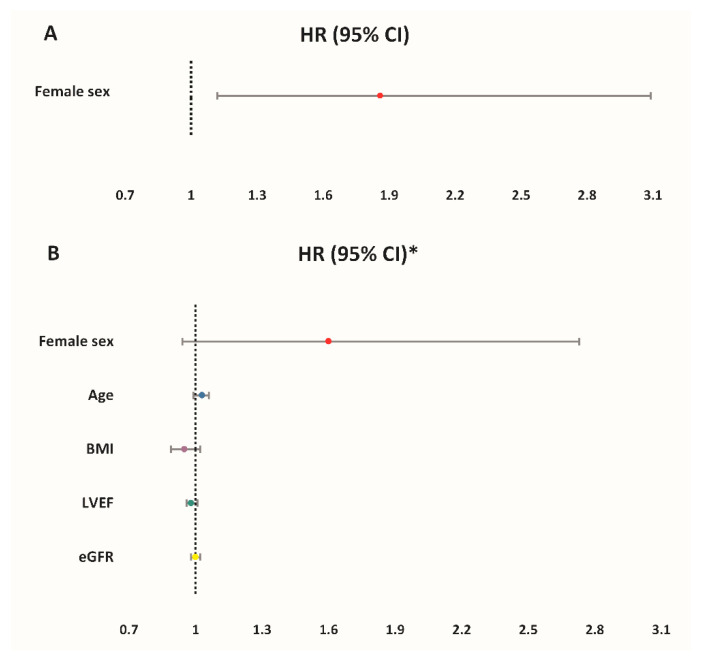
Hazard ratios (HR) of MACCE for different predictors: (**A**) Unadjusted rates; (**B**) Adjusted rates. * Multivariate Cox regression model-reference groups for categorical variables: male sex; no diabetes mellitus. Legend: MACCE, major adverse cardiovascular and cerebrovascular events (composite of cardiac mortality, ischemic stroke, urgent coronary revascularization and nonfatal myocardial infarction); LVEF, left ventricular ejection fraction; eGFR, estimated glomerular filtration rate; BMI, body mass index; RBC, red blood cells.

When looking at the female sex as a predictor variable for MACCE, it was significantly associated with an increased incidence of MACCE in the univariate analysis (HR 1.86, 95%CI 1.12–3.09, *p* = 0.014) (Figure 2A). However, this difference became statistically non-significant after the multivariable adjustment for age, LVEF, eGFR and BMI (HR 1.60, 95%CI 0.94–2.73, *p* = 0.083) (Figure 2B).

**Figure 3:**
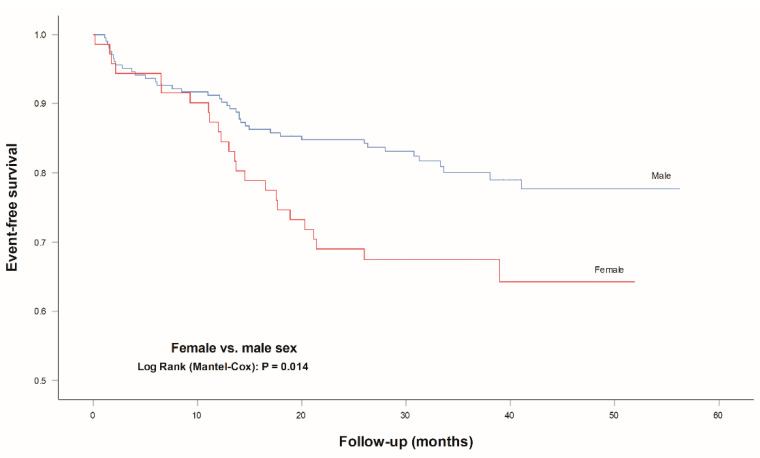
Event-free survival for MACCE according to sex. Legend: MACCE, major adverse cardiovascular and cerebrovascular events (composite of cardiac mortality, ischemic stroke, urgent coronary revascularization and nonfatal myocardial infarction).

The cumulative incidence of MACCE was higher in female patients with earlier MACCE occurrence during follow-up (38.71, 95%CI 34.31–43.10 vs. 47.33, 95%CI 44.83–49.82 months, *p* = 0.014) (Figure 3).

**Figure 4:**
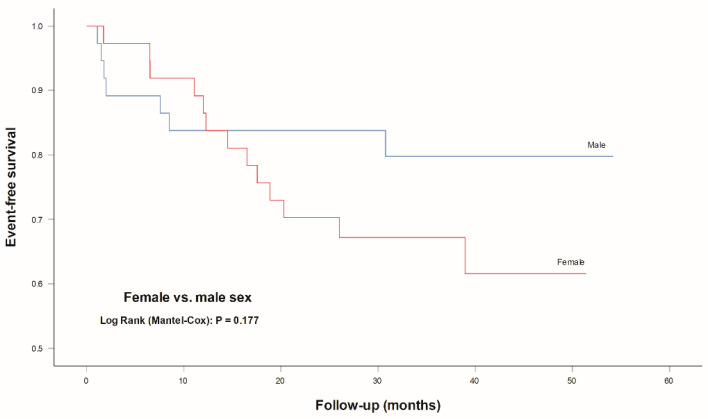
Event-free survival for MACCE according to sex in the propensity score matched cohort. Propensity score matching analysis with a nearest neighbor matching algorithm for age, BMI and eGFR (1:1 allocation and a caliper of 0.01). Legend: MACCE, major adverse cardiovascular and cerebrovascular events (composite of cardiac mortality, ischemic stroke, urgent coronary revascularization and nonfatal myocardial infarction).

However, when evaluating a propensity score matched cohort, there was no statistically significant difference in the cumulative incidence of MACCE (*p* = 0.177) (Table 3 and Figure 4).

**Figure 5:**
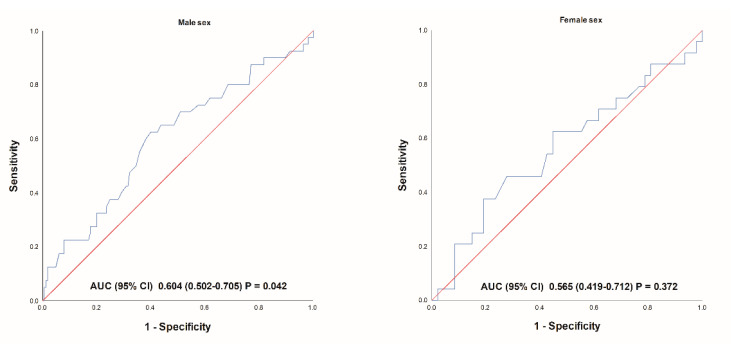
Receiver operating characteristic of predicting MACCE for GRACE score in different sex groups. Legend: MACCE, major adverse cardiovascular and cerebrovascular events (composite of cardiac mortality, ischemic stroke, urgent coronary revascularization and nonfatal myocardial infarction); GRACE, Global Registry of Acute Coronary Events.

When evaluating the performance of the GRACE score for the prediction of MACCE, it showed inferior AUC values in the female group compared to that in male patients (0.565, 95% CI 0.419–0.712, *p* = 0.372 vs. 0.604, 95% CI 0.502–0,705, *p* = 0.042, respectively) (Figure 5).

## 4. Discussion

The equality in the management and outcomes of NSTE-ACS patients has been aspired, and numerous studies have been conducted to reveal any sex-related disparities. However, due to population and study heterogeneity, the existing literature is still conflicting and lacks all-supporting data [15,16]. This study presents sex-disaggregated data of hospitalized NSTE-ACS patients undergoing a coronary angiography who were managed with all-treatment strategies, including PCI, CABG and conservative management. Importantly, this prospective study encompassed patients with a similar discharge therapy comorbidity burden, while attempting to control for other confounding variables.

The main findings of this study could be summarized. Firstly, the female patients were mostly older. Secondly, when evaluating the unadjusted rates, the female patients had a higher number of ischemic strokes, cardiac mortality and MACCE with significant discrepancy in the event-free survival curves. However, in the propensity score matched cohort, female patients did not have a statistically higher incidence of MACCE. Similarly, female sex was not a significant independent predictor of worse outcomes per se. Finally, the predictive strength of the GRACE score in female patients was inferior compared to in the male population.

Consistent with previous studies, the present analysis revealed that female NSTE-ACS patients are generally more prone to adverse outcomes. However, most previous studies had retrospective designs, potentially leading to selection bias and inaccurate data capture [10]. Several registral analyses reported big data on the sex-based differences in the management and outcomes of ACS patients, but the lack of pharmacotherapy data is worrisome due to its important impact on the outcomes. In addition, female patients are often older and have more comorbidities, which also influence the outcomes [10,17]. Finally, female patients show poorer adherence to medications, including premature disruption of dual antiplatelet or statin therapy [18,19], and often discontinue the cardiac rehabilitation program [20]. This could potentially explain the ~10-month delay in worse outcomes observed in this study.

Interestingly, Kumbhani et al. reported similar sex-based mortality in a PCI-treated NSTE-ACS cohort, but a significantly higher long-term mortality in younger and troponin-negative female patients [6,11]. However, when looking solely at the elderly NSTE-ACS population, female patients exhibited similar or better outcomes in a study by De Carlo et al. [12]. Nevertheless, older age was recognized as a negative predictor and mediator of worse outcomes in female patients. Additionally, female patients undergo less coronary revascularization during an NSTE-ACS event, but early revascularization has been shown to be a substantial predictor of better outcomes in these patients [12]. In fact, a routine invasive approach has been feasible and safe in women, leading to better outcomes [21]. 

When looking at the short-term outcomes, data from the J-PCI registry show that a PCI-treated female Japanese NSTE-ACS cohort had a higher rate of bleeding and other complications, but there was no statistically significant difference in the adjusted rates of in-hospital mortality [22]. Similarly, Song et al. reported similar sex-based in-hospital mortality after an NSTE-ACS event in the Chinese Registry of Acute Coronary Events (CRACE) registry [17].

A recent meta-analysis of Thrombolysis In Myocardial Infarction (TIMI) trials reported a higher comorbidity burden and a lower utilization of evidence-based treatment in female NSTE-ACS patients, but also a lower adjusted all-cause mortality among female patients. The mean follow-up in the included trials was ~23 months, which is similar (yet shorter) to the present study [15]. Another meta-analysis of observational studies by Wang et al. showed that female NSTE-ACS patients have a similar adjusted short-term and long-term mortality [16]. One of the longest follow-up studies by Vogel et al. did not show a significant sex-based difference in the adjusted 7-year mortality rates between female and male patients, but only ~50% patients underwent a coronary angiography [23]. Recent analysis from the National Inpatient Sample database showed a strong association between receipt of a coronary angiography and lower in-hospital mortality with an increasing trend in the utilization of coronary angiography among both sex categories [24]. Additionally, most prior studies did not include patients undergoing surgical revascularization [22,23]. One of the largest sex-specific randomized studies on invasively treated NSTE-ACS patients did not reveal independent association of female gender and worse 6-month outcomes after the multivariable adjustment [4]. Another propensity matched analysis showed a lower risk of long-term all-cause mortality and cardiovascular mortality in female patients [5]. Similarly, Bosch et al. reported better adjusted 30-month outcomes of 95 female patients after an NSTE-ACS event [25]. Alfredsson et al. also reported lower adjusted rates of 1-year mortality in female patients [7].

The preferred access site for invasive management is important as it has been shown that female patients particularly benefit from the radial approach, even after accounting for the smaller vessel diameter [26]. One should have in mind the predominant use of the femoral artery worldwide before 2010 and most older studies enrolled patients undergoing a femoral approach. The present study enrolled patients predominantly managed over radial access that were equally prescribed with guideline-directed medication therapy upon discharge. Sex-differences in the prescription of different antiplatelet regimes, and the utilization and optimization of guideline-directed medical therapy could influence the prognosis. Finally, this study involved a cohort equally managed with all-treatment strategies, as it has been previously proposed that worse outcomes in female NSTE-ACS patients could be attributed to a lower utilization of invasive management [10,12]. This study offers several strengths related to the encompassment of all-treatment strategies and the utilization of coronary angiography in all patients. Additionally, the enrolled NSTE-ACS population had relatively low risk features, as evident from the GRACE risk score, which are not sufficiently represented in the literature. While there is plenty of sex-based research on NSTE-ACS patients, the population and study heterogeneity implicate the need for more data. In fact, future multidimensional research evaluating sex, ethnicity, education and socioeconomic status should be encouraged to reduce the risk-treatment paradox that is present even in the contemporary era [24,27]. Awareness about heart disease in the female population should be encouraged. Healthcare workers should be sensibilized to timely recognize female patients with acute coronary syndrome. Access to healthcare, timely diagnosis, invasive management and evidence-based therapy are crucial in the equity of care of this heterogenous NSTE-ACS population. The aforementioned should motivate the timely healthcare measures for the global, equal and systematic approach to both female and male patients suffering from NSTE-ACS.

It is important to highlight several limitations of this study. While there was no difference in discharge therapy, the study coordinators did not record the patients’ adherence to the therapy during the follow-up. However, each of the specialists involved in the follow-up and treatment of these patients was dedicated to titrate and guide therapy, according to the relevant guidelines. Furthermore, this is a single center study that comprised a relatively small sample size and detected a low incidence of events, which could have affected the statistical analyses and prevented additional sub-analyses. As with other studies, the composite outcome in this study was not standardized, which impedes the study comparison. Finally, the study mostly encompassed low-to-medium risk NSTE-ACS patients without previous CAB; therefore, care should be taken before applying these results to a high-risk NSTE-ACS population with prior cardiac surgery. Similarly, the results are not applicable to patients with active malignancy, as they were excluded. Even though the treatment strategy was based on the Heart Team’s decision, a sex-related selection bias could not be fully eliminated. 

## 5. Conclusions

In conclusion, this study showed that female NSTE-ACS patients undergoing all-treatment strategies have significantly worse long-term outcomes. However, the observed disparities are dependent on the differences in baseline characteristics including age, BMI and kidney function. Future studies and global healthcare initiatives are encouraged to improve and equalize the care of the heterogenous NSTE-ACS population.

## Figures and Tables

**Table 1:** Comparison of the baseline characteristics.

Variables	Sex		*p* Value
	Female (*n* = 71)	Male (*n* = 205)	
Age (years)	69.0 (60.0–75.0)	63.0 (55.0–73.0)	0.008 *
BMI (kg/m^2^)	26.3 (23.9–29.4)	28.2 (26.1–30.9)	<0.001 *
NSTE-ACS subtype			0.444 †
NSTEMI	61 (85.9%)	168 (82.0%)	
UA	10 (14.1%)	37 (18.0%)	
Comorbidities			
Arterial hypertension	50 (70.4%)	127 (62.0%)	0.200 †
Diabetes mellitus	22 (31.0%)	61 (29.8%)	0.846 †
Hyperlipidemia	40 (56.3%)	98 (47.8%)	0.215 †
Chronic kidney disease	4 (5.6%)	8 (3.9%)	0.538 †
Family history of CV disease	21 (29.6%)	80 (39.0%)	0.154 †
Active smoking	27 (37.1%)	88 (42.9%)	0.481 †
Atrial fibrillation	8 (11.3%)	18 (8.8%)	0.536 †
Prior MI	8 (11.4%)	22 (10.7%)	0.872 †
Prior PCI	8 (11.3%)	15 (7.3%)	0.299 †
Prior CVI	5 (7.0%)	7 (3.4%)	0.196 †
Prior AAA	1 (1.4%)	9 (4.4%)	0.496 †
COPD	3 (4.2%)	30 (14.6%)	0.053 †
Anemia	12 (16.9%)	25 (12.2%)	0.316 †
Laboratory parameters			
Red blood cells	4.46 ± 0.42	4.81 ± 0.70	<0.001 ‡
Hgb (g/L)	132.13 ± 13.39	144.39 ± 14.86	<0.001 ‡
RDW	13.94 ± 2.26	14.06 ± 5.78	0.873 ‡
Platelets	255.13 ± 68.14	224.03 ± 58.73	<0.001 ‡
White blood cells	9.28 ± 2.60	9.64 ± 2.90	0.355 ‡
Glucose (mmol/L)	8.29 ± 3.03	8.96 ± 7.11	0.440 ‡
CRP (mmol/L)	11.25 ± 20.37	21.89 ± 47.40	0.151 ‡
eGFR (mL/min)	76.44 ± 22.43	94.04 ± 27.91	<0.001 ‡
Echocardiography			
LVEF (%)	60.38 ± 10.12	57.76 ± 10.10	0.060 ‡
Left atrial diameter	3.90 ± 1.00	4.26 ± 0.76	0.006 ‡
ECG changes			0.998 †
T-wave changes	37 (52.9%)	108 (53.2%)	
ST depression	21 (30.0%)	60 (29.6%)	
Risk scores			
GRACE 2.0 score	103.99 ± 28.65	99.39 ± 26.63	0.220 ‡
TIMI score	2.58 ± 0.72	2.40 ± 0.94	0.149 ‡
Killip class > 1	10 (14.1%)	17 (8.3%)	0.236 †
Follow-up (months)	30.1 (19.6–40.3)	33.1 (26.1–42.8)	0.083 *

Data are expressed as mean ± SD, number (percent) or median (interquartile range). * Mann–Whitney U test; † Chi-square test; ‡ Student’s *t*-test. Abbreviations: AAA, abdominal aortic aneurysm; BMI, body mass index; CAD, coronary arterial disease; COPD, chronic obstructive pulmonary disease; CRP, C-reactive peptide; CV, cardiovascular; CVI, cerebrovascular insult; eGFR, estimated glomerular filtration rate; GRACE, Global Registry for Acute Coronary Events; Hgb, hemoglobin; LVEF, left ventricular ejection fraction; MI, myocardial infarction; NSTE-ACS, non-ST elevation acute coronary syndrome; NSTEMI, non ST segment elevation myocardial infarction; UA, unstable angina; PCI, percutaneous coronary intervention; RDW, red cell distribution width; TIMI, Thrombolysis in Myocardial Infarction.

**Table 2:** Treatment and angiographic characteristics.

Variables	Sex		*p* Value
	Female (*n* = 71)	Male (*n* = 205)	
Access site			0.129 *
Radial	63 (88.7%)	193 (94.1%)	
Femoral	8 (11.3%)	12 (5.9%)	
Treatments			0.271 *
Conservative	21 (29.6%)	42 (20.5%)	
PCI	27 (38.0%)	83 (40.5%)	
CABG	23 (32.4%)	80 (39.0%)	
Coronary angiogram			
Left main disease	10 (14.1%)	22 (10.7%)	0.447 *
Single-vessel disease	38 (53.6%)	83 (40.5%)	0.258 *
Two-vessel disease	16 (22.5%)	40 (19.5%)	0.585 *
Three-vessel disease	17 (23.9%)	82 (40.0%)	0.177 *
Culprit artery			0.063 *
Left main	10 (14.1%)	22 (10.7%)	
Left anterior descending	29 (40.8%)	87 (42.4%)	
Circumflex	13 (18.3%)	46 (22.4%)	
Right coronary	19 (26.8%)	50 (24.4%)	
Bifurcation lesion	13 (18.3%)	25 (12.2%)	0.197 *
Coronary calcification	3 (4.2%)	18 (8.8%)	0.212 *
Discharge therapy			
Beta blockers	57 (80.3%)	143 (69.8%)	0.087 *
ACE-I/ARB	46 (64.8%)	130 (63.4%)	0.836 *
ASA	66 (93.0%)	193 (94.1%)	0.720 *
P2Y12 inhibitors	56 (78.9%)	176 (85.9%)	0.166 *
Statins	63 (88.7%)	189 (92.2%)	0.372 *
Calcium channel blockers	12 (16.9%)	23 (11.3%)	0.220 *

Data are expressed as number (percent). * Chi-square test. Abbreviations: ACE-I, angiotensin converting enzyme inhibitors; ARB, angiotensin receptor blockers; ASA, acetylsalicylic acid; CABG, coronary artery bypass grafting; PCI, percutaneous coronary intervention.

**Table 3:** Adverse events during the study period.

Variables	Sex		*p* Value
	Female (*n* = 37)	Male (*n* = 37)	
Age (years)	69.0 (60.0–75.0)	63.0 (55.0–73.0)	0.673 *
BMI (kg/m^2^)	26.3 (23.9–29.4)	28.2 (26.1–30.9)	0.804 *
eGFR (mL/min)	76.44 ± 22.43	94.04 ± 27.91	0.970 *
Comorbidities			
Arterial hypertension	24 (64.9%)	27 (73.0%)	0.451 †
Diabetes mellitus	13 (35.1%)	12 (32.4%)	0.806 †
Hyperlipidemia	17 (45.9%)	23 (62.2%)	0.162 †
Active smoking	6 (16.2%)	10 (27.0%)	0.733 †
Atrial fibrillation	3 (8.1%)	3 (8.1%)	1.000 ‡
Anemia	3 (8.1%)	7 (18.9%)	0.174 ‡

Propensity score matching analysis with a nearest neighbor matching algorithm for age, BMI and eGFR (1:1 allocation and a caliper of 0.01). Data are expressed as number (percent) or median (interquartile range). * Mann–Whitney U test; † Chi-square test; ‡ Fisher’s test. Abbreviations: BMI, body mass index; eGFR, estimated glomerular filtration rate.

## Data Availability

Data related to the study are available upon request to the corresponding author.

## Supplementary Materials

The following are available online at https://www.mdpi.com/article/10.3390/jcm10132802/s1, Supplementary Figure S1: Flow diagram of the study.
