# Supplementary material for: Sex Differences in Characteristics and Outcomes among Low-Risk Non-ST-Elevation Acute Coronary Syndrome Patients during Long Term Follow-Up
Source: J Clin Med. 2021 Jun 25;10(13):2802. doi: 10.3390/jcm10132802 (PMC8267884; doi:10.3390/jcm10132802)
Supplement: Supplementary file 1 [file jcm-10-02802-s001.zip › jcm-1263672-supplementary.pdf]

Supplementary Figures:

Supplementary Figure S1. Flow diagram of the study.

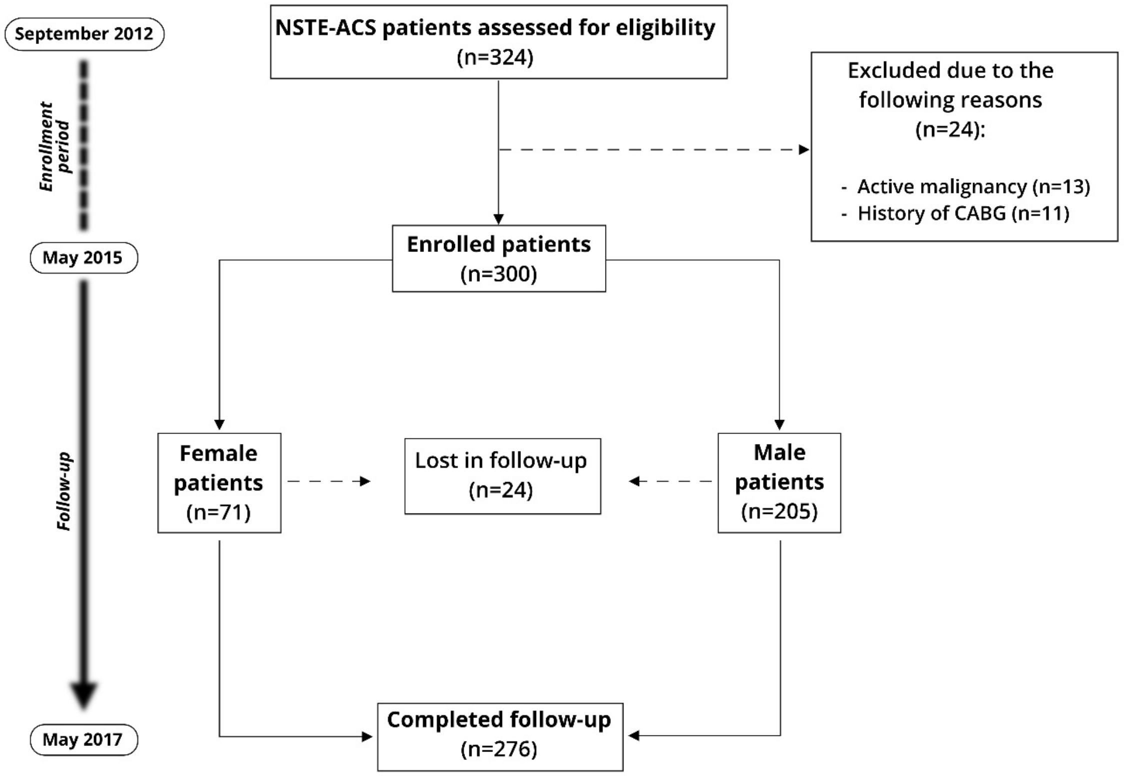

Legend: NSTE-ACS – non-ST-elevation acute coronary syndrome.
